# P05.55. Qualitative findings from piloting the LEAP Project – an online spirituality-based depression intervention for young adults

**DOI:** 10.1186/1472-6882-12-S1-P415

**Published:** 2012-06-12

**Authors:** A Kania, S Moritz, S Malhotra, R Maser, B Rickhi, J Toews

**Affiliations:** 1 University of Calgary, Calgary, Alberta, Canada; 2 Canadian Institute of Natural and Integrative Medicine (CINIM), Calgary, Canada

## Purpose

To report preliminary qualitative findings from a clinical trial that piloted an 8-week, online, spirituality-based depression intervention called the LEAP Project (www.leapproject.com) for young adults with depression. A qualitative approach was used in order to gain a more in-depth understanding of the intervention impact on study participants.

## Methods

Participants were recruited using a convenience sampling strategy and invited to partake in an in-depth, semi-structured interview upon completion of the intervention. The LEAP Project intervention consists of eight modules (1) Consciousness and Self Acceptance, (2) Appreciation of Beauty & Creativity, (3). Mystery and Meaning, (4) Gratitude, (5) Compassion, (6) Acceptance, (7) Forgiveness, and (8) Celebration. Each is presented using video clips, music, visualizations, true stories and life practices. All interviews were digitally recorded and transcribed verbatim. Data were analyzed using a descriptive qualitative content analysis approach. Transcripts were read and re-read, and coded to preliminary categories and emerging themes of substantive meanings through an iterative process.

## Results

The impact of the intervention on participants was multi-faceted. Participants reflected that the intervention was grounding and led to changed perspectives of others, themselves, and their surroundings. Participants experienced a greater sense of control over emotions and resulting behaviors, as well as a sense of connection and realization that they are not alone. The latter was identified as a key element in their ability to cope with depression. Participants described an improvement in well-being characterized by: higher energy levels, increased motivation, reduced negative feelings such as fear, anger, frustration, and reduced feelings of depression.

## Conclusion

Our findings suggest that the LEAP Project impacts young adults with depression by shifting perspectives of life situations and others, providing a sense of control and connection, improving coping abilities and enhancing well-being. Such a program may be a useful and valuable resource in the treatment of depression.

